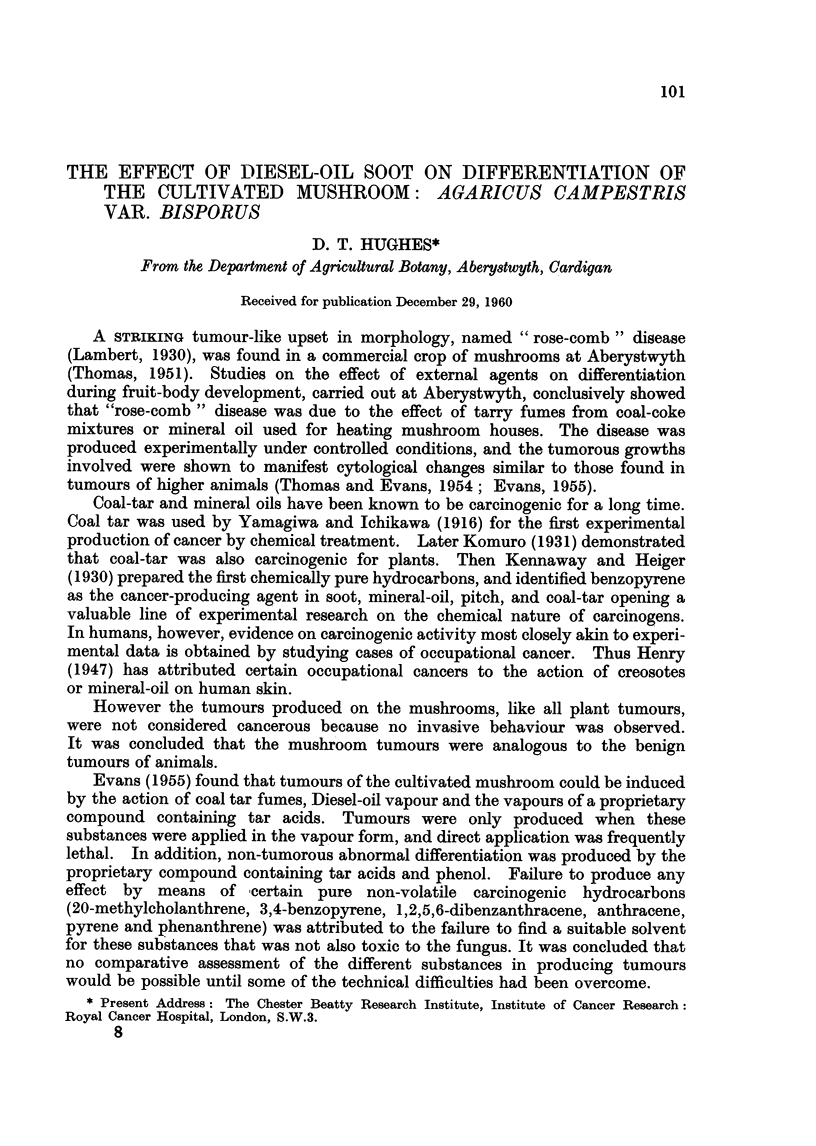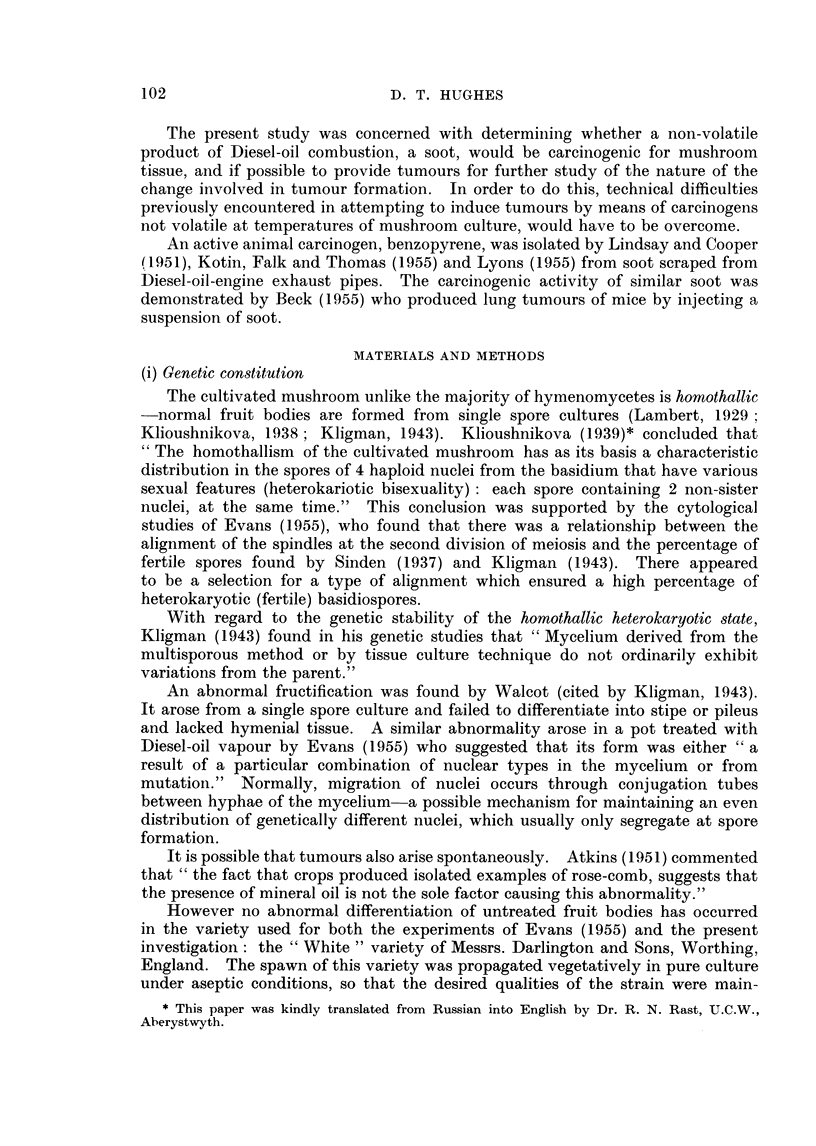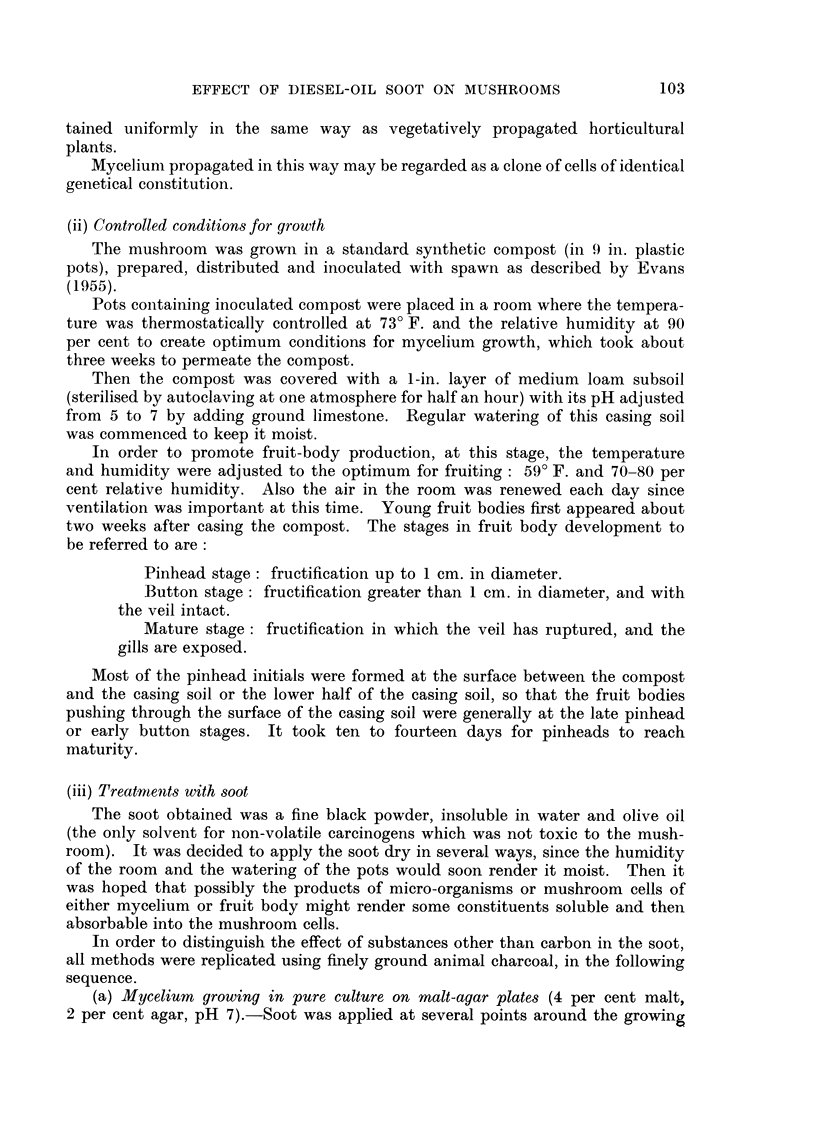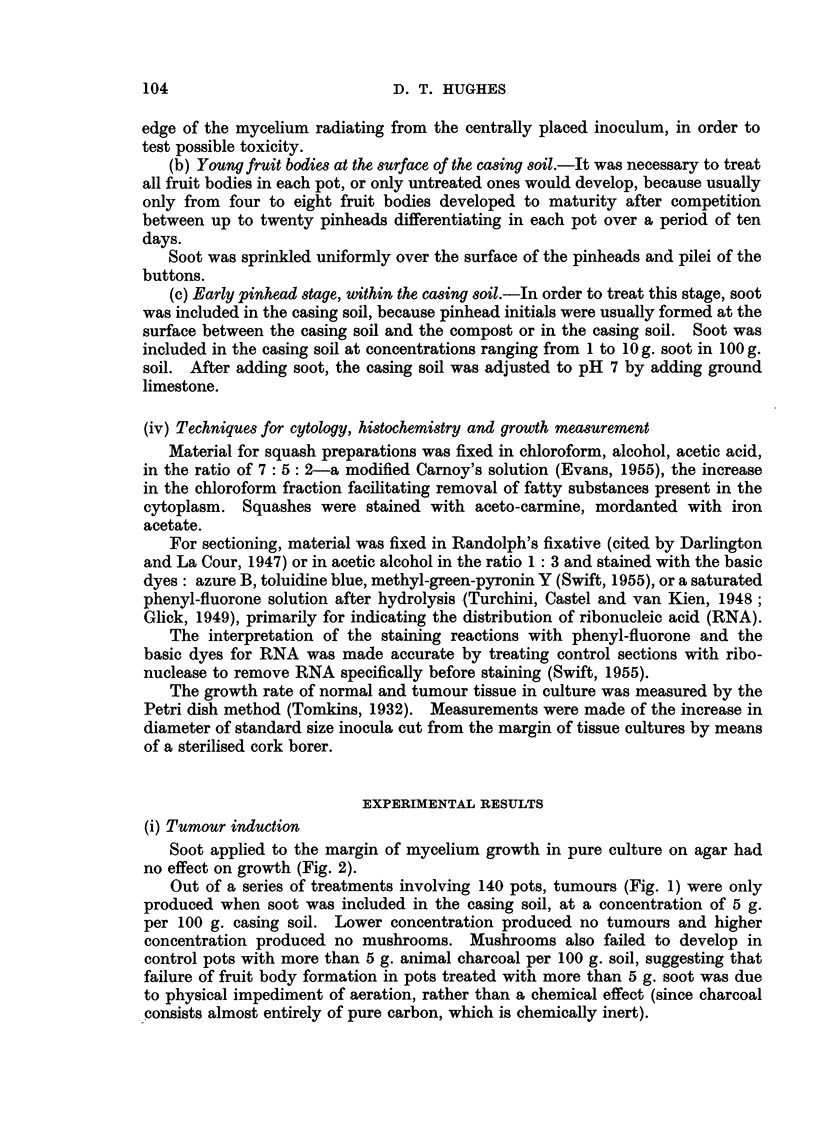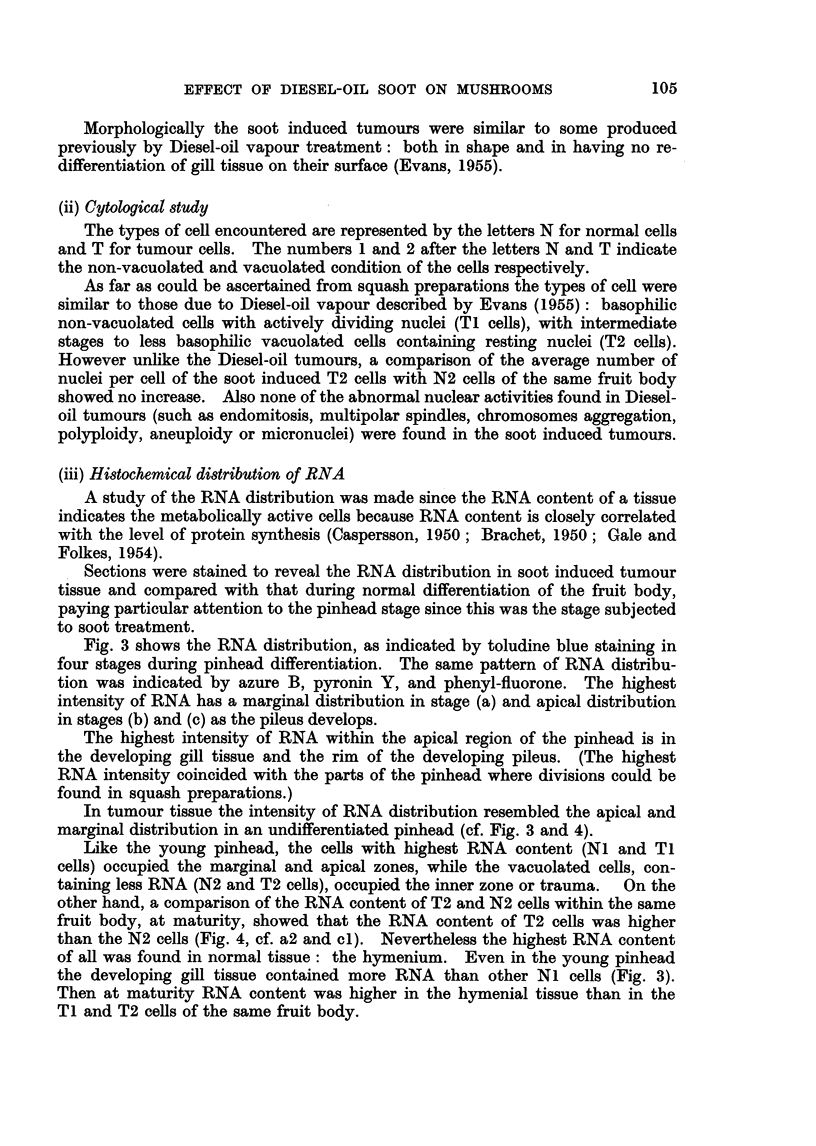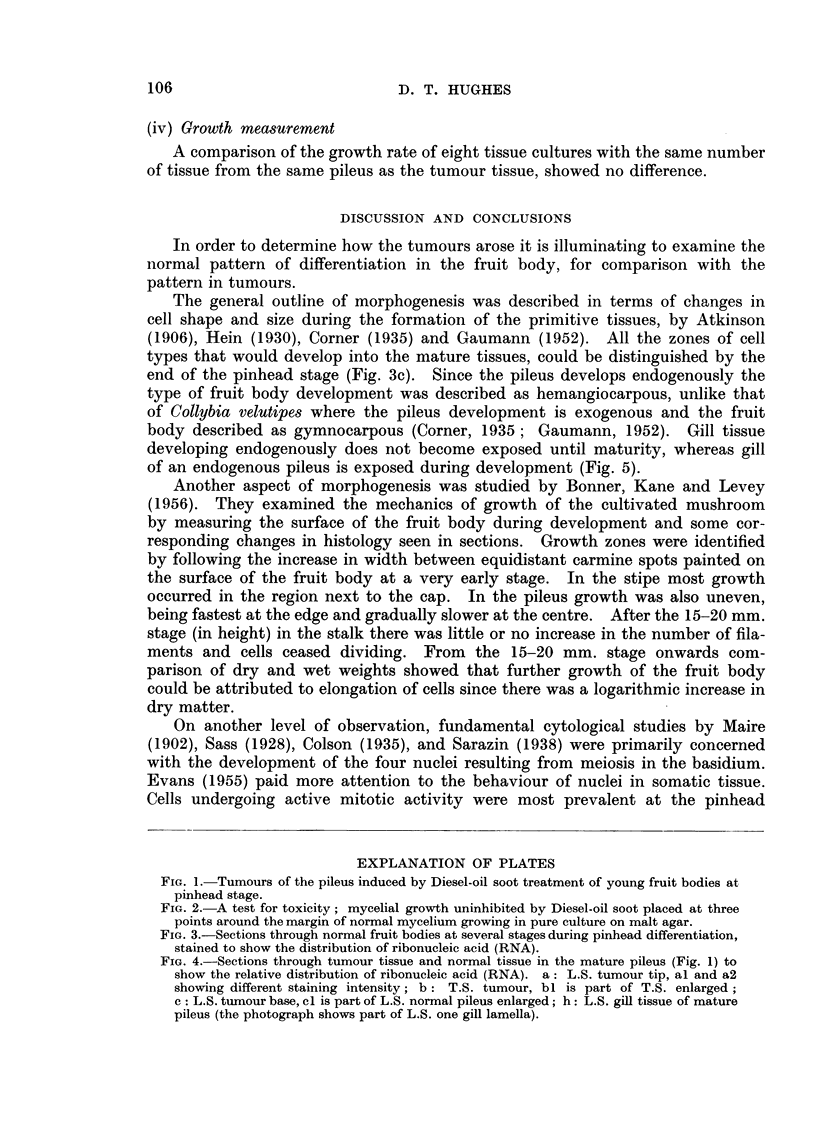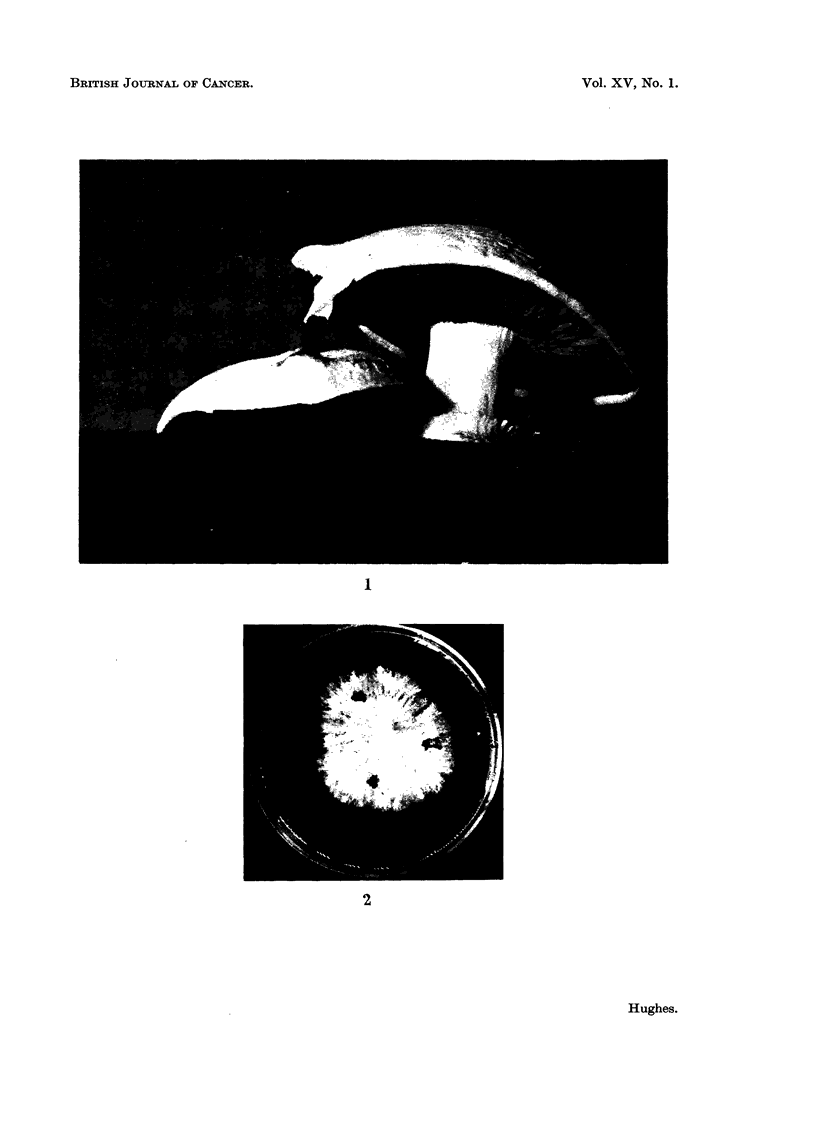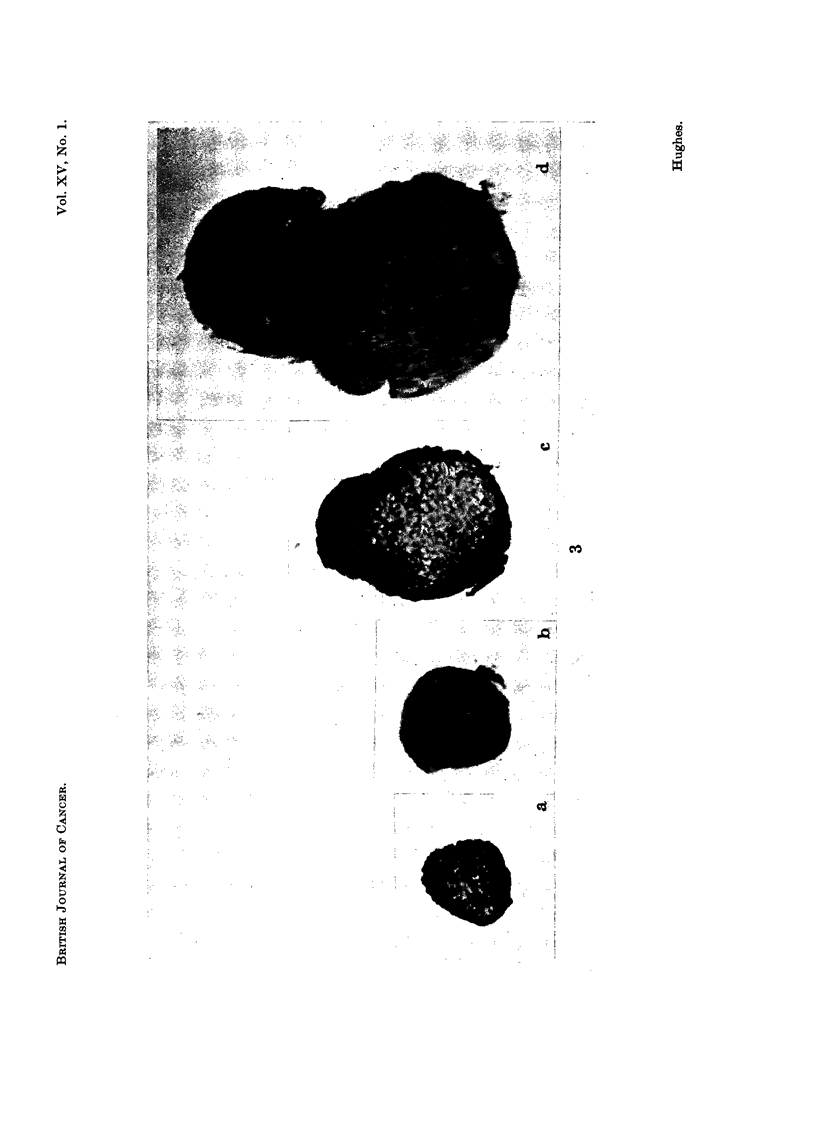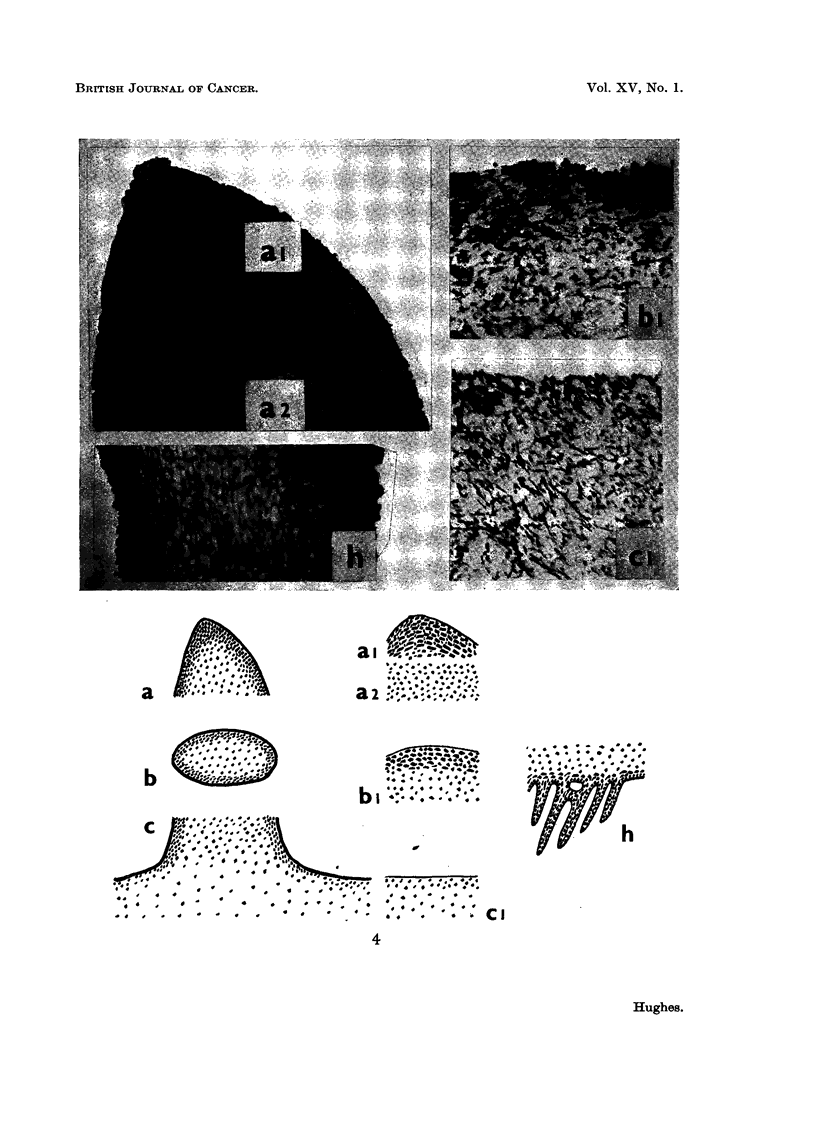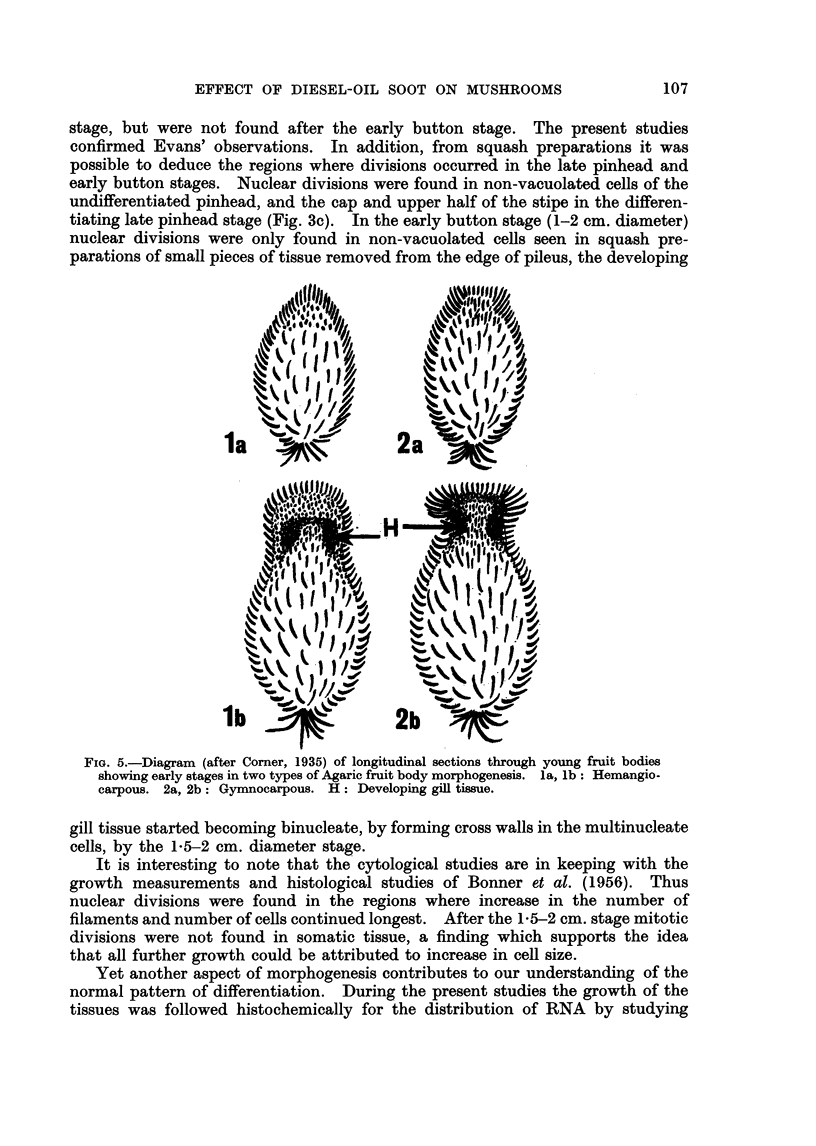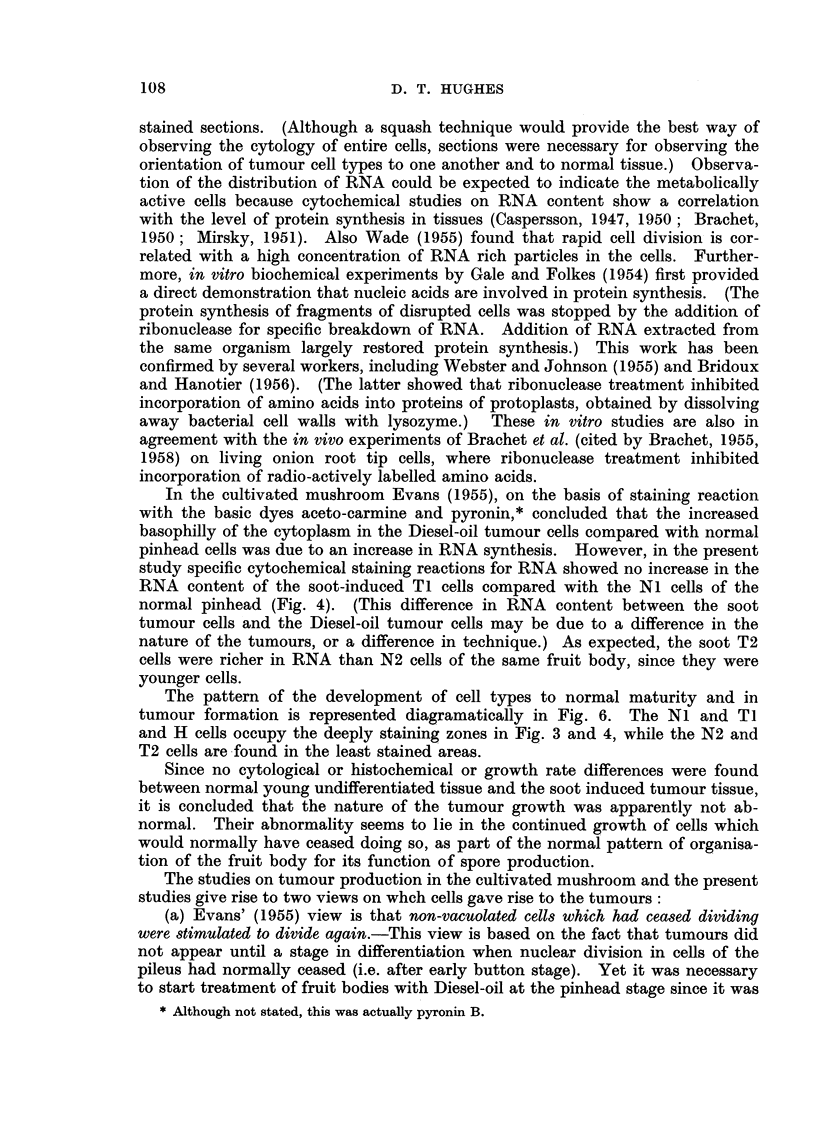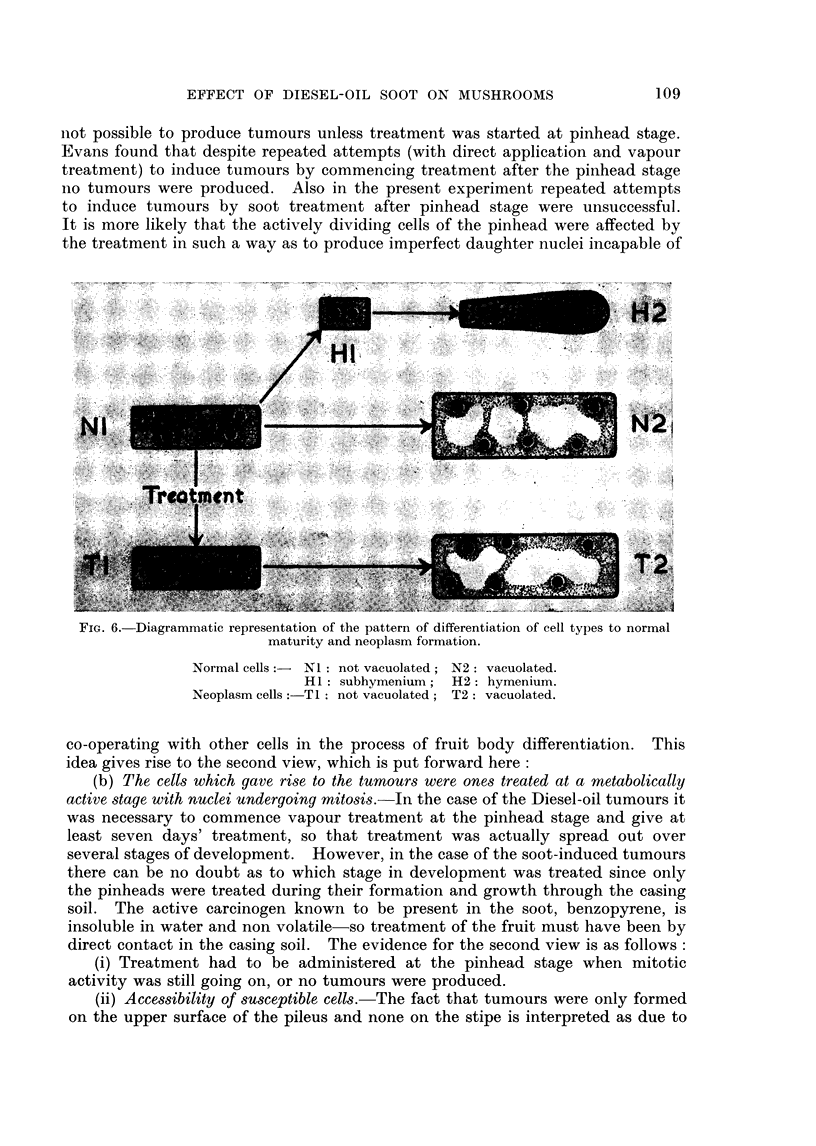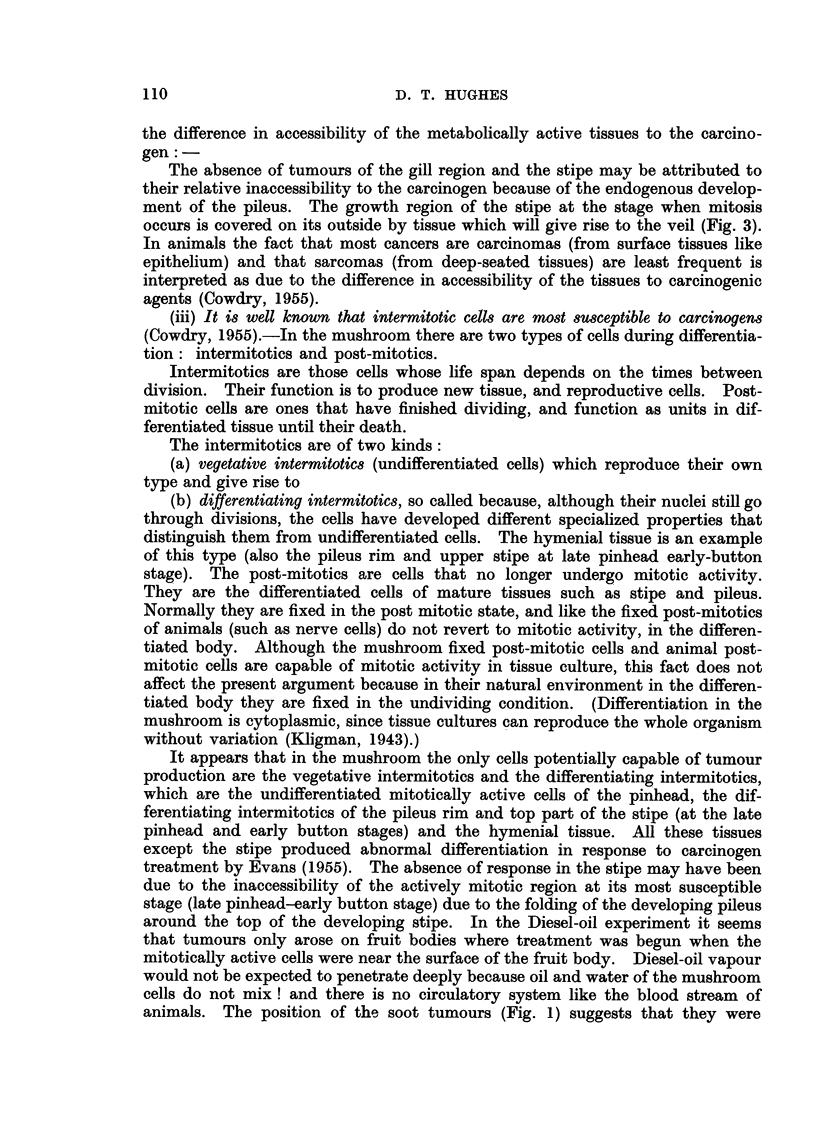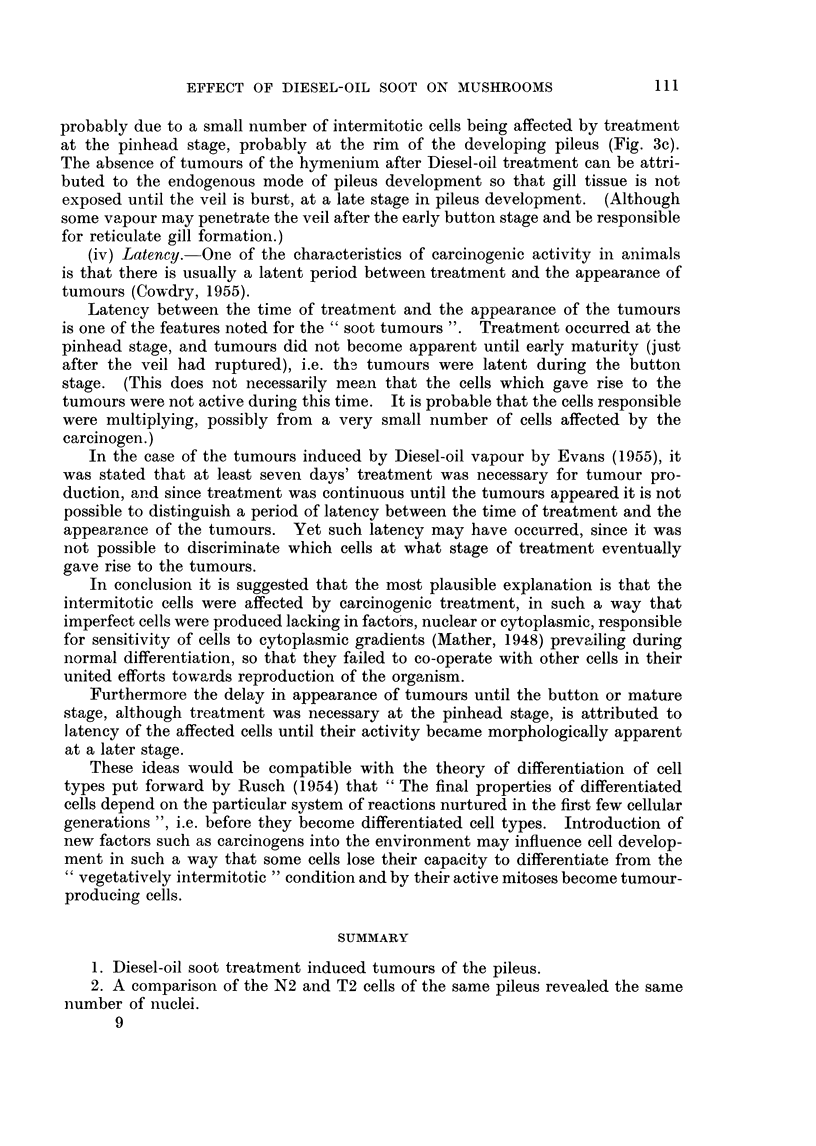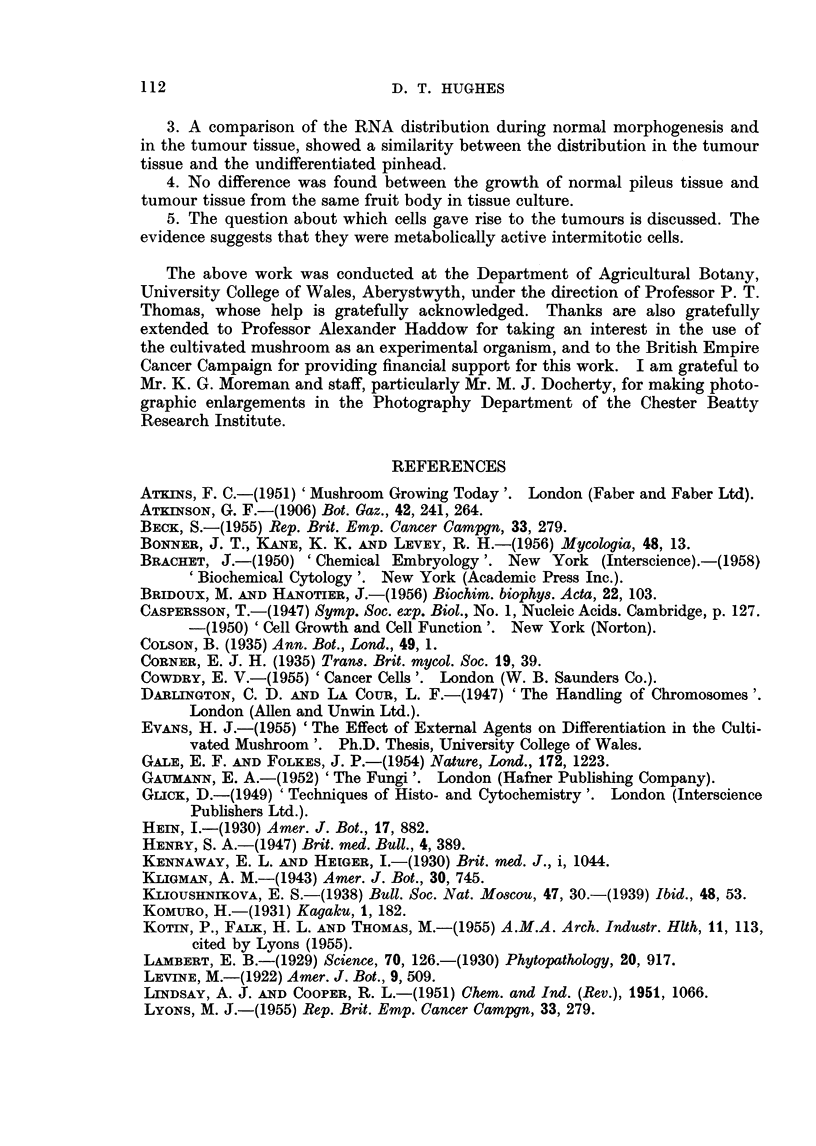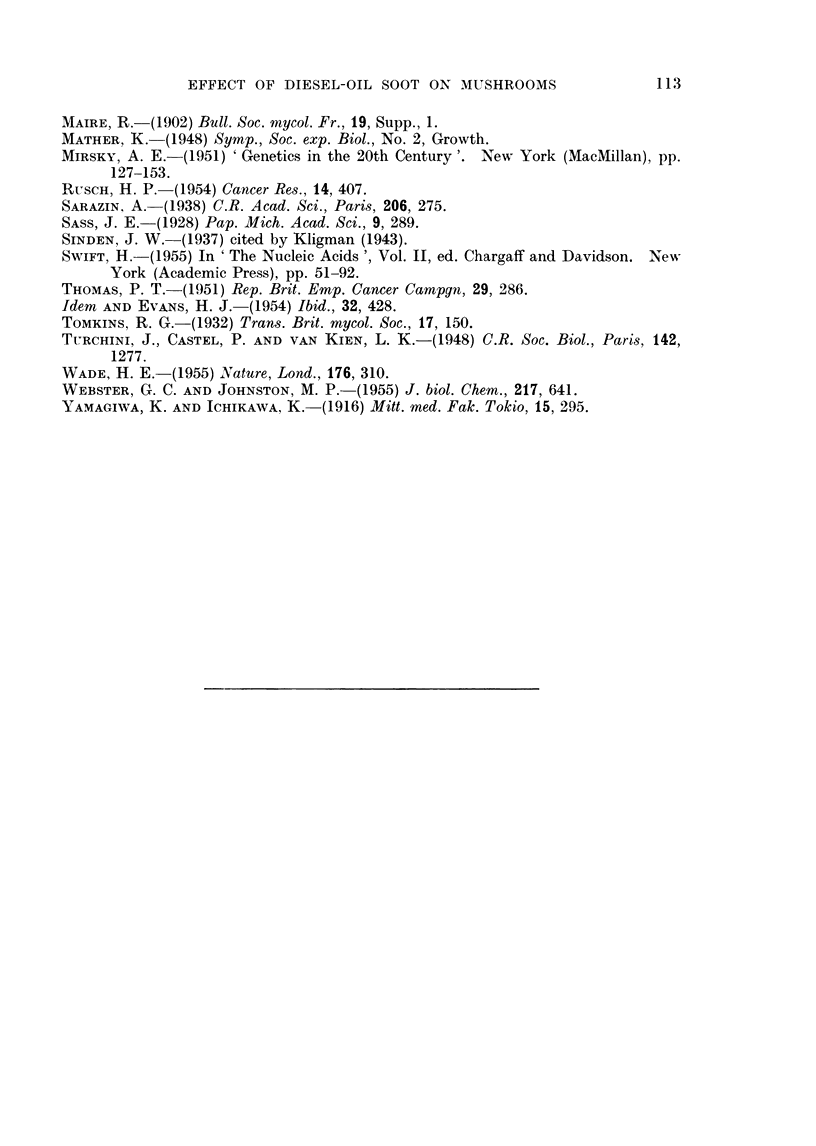# The Effect of Diesel-Oil Soot on Differentiation of the Cultivated Mushroom: Agaricus Campestris Var. Bisporus

**DOI:** 10.1038/bjc.1961.11

**Published:** 1961-03

**Authors:** D. T. Hughes

## Abstract

**Images:**


### THE EFFECT OF DIESEL-OIL SOOT ON DIFFERENTIATION OF THE CULTIVATED MUSHROOM: *AGARICUS CAMPESTRIS* VAR. *BISPORUS*

D. T. HUGHES*

*From the Department of Agricultural Botany, Aberystwyth, Cardigan*

Received for publication December 29, 1960

A STRIKING tumour-like upset in morphology, named " rose-comb " disease (Lambert, 1930), was found in a commercial crop of mushrooms at Aberystwyth (Thomas, 1951). Studies on the effect of external agents on differentiation during fruit-body development, carried out at Aberystwyth, conclusively showed that "rose-comb " disease was due to the effect of tarry fumes from coal-coke mixtures or mineral oil used for heating mushroom houses. The disease was produced experimentally under controlled conditions, and the tumorous growths involved were shown to manifest cytological changes similar to those found in tumours of higher animals (Thomas and Evans, 1954 ; Evans, 1955).

Coal-tar and mineral oils have been known to be carcinogenic for a long time. Coal tar was used by Yamagiwa and Ichikawa (1916) for the first experimental production of cancer by chemical treatment. Later Komuro (1931) demonstrated that coal-tar was also carcinogenic for plants. Then Kennaway and Heiger (1930) prepared the first chemically pure hydrocarbons, and identified benzopyrene as the cancer-producing agent in soot, mineral-oil, pitch, and coal-tar opening a valuable line of experimental research on the chemical nature of carcinogens. In humans, however, evidence on carcinogenic activity most closely akin to experimental data is obtained by studying cases of occupational cancer. Thus Henry (1947) has attributed certain occupational cancers to the action of creosotes or mineral-oil on human skin.

However the tumours produced on the mushrooms, like all plant tumours, were not considered cancerous because no invasive behaviour was observed. It was concluded that the mushroom tumours were analogous to the benign tumours of animals.

Evans (1955) found that tumours of the cultivated mushroom could be induced by the action of coal tar fumes, Diesel-oil vapour and the vapours of a proprietary compound containing tar acids. Tumours were only produced when these substances were applied in the vapour form, and direct application was frequently lethal. In addition, non-tumorous abnormal differentiation was produced by the proprietary compound containing tar acids and phenol. Failure to produce any effect by means of certain pure non-volatile carcinogenic hydrocarbons (20-methylcholanthrene, 3,4-benzopyrene, 1,2,5,6-dibenzanthracene, anthracene, pyrene and phenanthrene) was attributed to the failure to find a suitable solvent for these substances that was not also toxic to the fungus. It was concluded that no comparative assessment of the different substances in producing tumours would be possible until some of the technical difficulties had been overcome.

* Present Address : The Chester Beatty Research Institute, Institute of Cancer Research : Royal Cancer Hospital, London, S.W.3.

The present study was concerned with determining whether a non-volatile product of Diesel-oil combustion, a soot, would be carcinogenic for mushroom tissue, and if possible to provide tumours for further study of the nature of the change involved in tumour formation. In order to do this, technical difficulties previously encountered in attempting to induce tumours by means of carcinogens not volatile at temperatures of mushroom culture, would have to be overcome.

An active animal carcinogen, benzopyrene, was isolated by Lindsay and Cooper (1951), Kotin, Falk and Thomas (1955) and Lyons (1955) from soot scraped from Diesel-oil-engine exhaust pipes. The carcinogenic activity of similar soot was demonstrated by Beck (1955) who produced lung tumours of mice by injecting a suspension of soot.

### MATERIALS AND METHODS

### (i) *Genetic constitution*

The cultivated mushroom unlike the majority of hymenomycetes is *homothallic* —normal fruit bodies are formed from single spore cultures (Lambert, 1929 ; Klioushnikova, 1938 ; Kligman, 1943). Klioushnikova (1939)* concluded that " The homothallism of the cultivated mushroom has as its basis a characteristic distribution in the spores of 4 haploid nuclei from the basidium that have various sexual features (heterokariotic bisexuality) : each spore containing 2 non-sister nuclei, at the same time." This conclusion was supported by the cytological studies of Evans (1955), who found that there was a relationship between the alignment of the spindles at the second division of meiosis and the percentage of fertile spores found by Sinden (1937) and Kligman (1943). There appeared to be a selection for a type of alignment which ensured a high percentage of heterokaryotic (fertile) basidiospores.

With regard to the genetic stability of the *homothallic heterokaryotic state*, Kligman (1943) found in his genetic studies that " Mycelium derived from the multisporous method or by tissue culture technique do not ordinarily exhibit variations from the parent."

An abnormal fructification was found by Walcot (cited by Kligman, 1943). It arose from a single spore culture and failed to differentiate into stipe or pileus and lacked hymenial tissue. A similar abnormality arose in a pot treated with Diesel-oil vapour by Evans (1955) who suggested that its form was either " a result of a particular combination of nuclear types in the mycelium or from mutation." Normally, migration of nuclei occurs through conjugation tubes between hyphae of the mycelium—a possible mechanism for maintaining an even distribution of genetically different nuclei, which usually only segregate at spore formation.

It is possible that tumours also arise spontaneously. Atkins (1951) commented that " the fact that crops produced isolated examples of rose-comb, suggests that the presence of mineral oil is not the sole factor causing this abnormality."

However no abnormal differentiation of untreated fruit bodies has occurred in the variety used for both the experiments of Evans (1955) and the present investigation : the " White " variety of Messrs. Darlington and Sons, Worthing, England. The spawn of this variety was propagated vegetatively in pure culture under aseptic conditions, so that the desired qualities of the strain were main-

* This paper was kindly translated from Russian into English by Dr. R. N. Rast, U.C.W., Aberystwyth.

tained uniformly in the same way as vegetatively propagated horticultural plants.

Mycelium propagated in this way may be regarded as a clone of cells of identical genetical constitution.

(ii) *Controlled conditions for growth*

The mushroom was grown in a standard synthetic compost (in 9 in. plastic pots), prepared, distributed and inoculated with spawn as described by Evans (1955).

Pots containing inoculated compost were placed in a room where the temperature was thermostatically controlled at 73° F. and the relative humidity at 90 per cent to create optimum conditions for mycelium growth, which took about three weeks to permeate the compost.

Then the compost was covered with a 1-in. layer of medium loam subsoil (sterilised by autoclaving at one atmosphere for half an hour) with its pH adjusted from 5 to 7 by adding ground limestone. Regular watering of this casing soil was commenced to keep it moist.

In order to promote fruit-body production, at this stage, the temperature and humidity were adjusted to the optimum for fruiting : 59° F. and 70–80 per cent relative humidity. Also the air in the room was renewed each day since ventilation was important at this time. Young fruit bodies first appeared about two weeks after casing the compost. The stages in fruit body development to be referred to are :

Pinhead stage : fructification up to 1 cm. in diameter.

Button stage : fructification greater than 1 cm. in diameter, and with the veil intact.

Mature stage : fructification in which the veil has ruptured, and the gills are exposed.

Most of the pinhead initials were formed at the surface between the compost and the casing soil or the lower half of the casing soil, so that the fruit bodies pushing through the surface of the casing soil were generally at the late pinhead or early button stages. It took ten to fourteen days for pinheads to reach maturity.

(iii) *Treatments with soot*

The soot obtained was a fine black powder, insoluble in water and olive oil (the only solvent for non-volatile carcinogens which was not toxic to the mushroom). It was decided to apply the soot dry in several ways, since the humidity of the room and the watering of the pots would soon render it moist. Then it was hoped that possibly the products of micro-organisms or mushroom cells of either mycelium or fruit body might render some constituents soluble and then absorbable into the mushroom cells.

In order to distinguish the effect of substances other than carbon in the soot, all methods were replicated using finely ground animal charcoal, in the following sequence.

(a) *Mycelium growing in pure culture on malt-agar plates* (4 per cent malt, 2 per cent agar, pH 7).—Soot was applied at several points around the growing edge of the mycelium radiating from the centrally placed inoculum, in order to test possible toxicity.

(b) *Young fruit bodies at the surface of the casing soil.*—It was necessary to treat all fruit bodies in each pot, or only untreated ones would develop, because usually only from four to eight fruit bodies developed to maturity after competition between up to twenty pinheads differentiating in each pot over a period of ten days.

Soot was sprinkled uniformly over the surface of the pinheads and pilei of the buttons.

(c) *Early pinhead stage, within the casing soil.*—In order to treat this stage, soot was included in the casing soil, because pinhead initials were usually formed at the surface between the casing soil and the compost or in the casing soil. Soot was included in the casing soil at concentrations ranging from 1 to 10 g. soot in 100 g. soil. After adding soot, the casing soil was adjusted to pH 7 by adding ground limestone.

(iv) *Techniques for cytology, histochemistry and growth measurement*

Material for squash preparations was fixed in chloroform, alcohol, acetic acid, in the ratio of 7 : 5 : 2—a modified Carnoy's solution (Evans, 1955), the increase in the chloroform fraction facilitating removal of fatty substances present in the cytoplasm. Squashes were stained with aceto-carmine, mordanted with iron acetate.

For sectioning, material was fixed in Randolph's fixative (cited by Darlington and La Cour, 1947) or in acetic alcohol in the ratio 1 : 3 and stained with the basic dyes : azure B, toluidine blue, methyl-green-pyronin Y (Swift, 1955), or a saturated phenyl-fluorone solution after hydrolysis (Turchini, Castel and van Kien, 1948 ; Glick, 1949), primarily for indicating the distribution of ribonucleic acid (RNA).

The interpretation of the staining reactions with phenyl-fluorone and the basic dyes for RNA was made accurate by treating control sections with ribonuclease to remove RNA specifically before staining (Swift, 1955).

The growth rate of normal and tumour tissue in culture was measured by the Petri dish method (Tomkins, 1932). Measurements were made of the increase in diameter of standard size inocula cut from the margin of tissue cultures by means of a sterilised cork borer.

### EXPERIMENTAL RESULTS

(i) *Tumour induction*

Soot applied to the margin of mycelium growth in pure culture on agar had no effect on growth (Fig. 2).

Out of a series of treatments involving 140 pots, tumours (Fig. 1) were only produced when soot was included in the casing soil, at a concentration of 5 g. per 100 g. casing soil. Lower concentration produced no tumours and higher concentration produced no mushrooms. Mushrooms also failed to develop in control pots with more than 5 g. animal charcoal per 100 g. soil, suggesting that failure of fruit body formation in pots treated with more than 5 g. soot was due to physical impediment of aeration, rather than a chemical effect (since charcoal consists almost entirely of pure carbon, which is chemically inert).

Morphologically the soot induced tumours were similar to some produced previously by Diesel-oil vapour treatment: both in shape and in having no re-differentiation of gill tissue on their surface (Evans, 1955).

(ii) *Cytological study*

The types of cell encountered are represented by the letters N for normal cells and T for tumour cells. The numbers 1 and 2 after the letters N and T indicate the non-vacuolated and vacuolated condition of the cells respectively.

As far as could be ascertained from squash preparations the types of cell were similar to those due to Diesel-oil vapour described by Evans (1955): basophilic non-vacuolated cells with actively dividing nuclei (T1 cells), with intermediate stages to less basophilic vacuolated cells containing resting nuclei (T2 cells). However unlike the Diesel-oil tumours, a comparison of the average number of nuclei per cell of the soot induced T2 cells with N2 cells of the same fruit body showed no increase. Also none of the abnormal nuclear activities found in Diesel-oil tumours (such as endomitosis, multipolar spindles, chromosomes aggregation, polyploidy, aneuploidy or micronuclei) were found in the soot induced tumours.

(iii) *Histochemical distribution of RNA*

A study of the RNA distribution was made since the RNA content of a tissue indicates the metabolically active cells because RNA content is closely correlated with the level of protein synthesis (Caspersson, 1950; Brachet, 1950; Gale and Folkes, 1954).

Sections were stained to reveal the RNA distribution in soot induced tumour tissue and compared with that during normal differentiation of the fruit body, paying particular attention to the pinhead stage since this was the stage subjected to soot treatment.

Fig. 3 shows the RNA distribution, as indicated by toludine blue staining in four stages during pinhead differentiation. The same pattern of RNA distribution was indicated by azure B, pyronin Y, and phenyl-fluorone. The highest intensity of RNA has a marginal distribution in stage (a) and apical distribution in stages (b) and (c) as the pileus develops.

The highest intensity of RNA within the apical region of the pinhead is in the developing gill tissue and the rim of the developing pileus. (The highest RNA intensity coincided with the parts of the pinhead where divisions could be found in squash preparations.)

In tumour tissue the intensity of RNA distribution resembled the apical and marginal distribution in an undifferentiated pinhead (cf. Fig. 3 and 4).

Like the young pinhead, the cells with highest RNA content (N1 and T1 cells) occupied the marginal and apical zones, while the vacuolated cells, containing less RNA (N2 and T2 cells), occupied the inner zone or trauma. On the other hand, a comparison of the RNA content of T2 and N2 cells within the same fruit body, at maturity, showed that the RNA content of T2 cells was higher than the N2 cells (Fig. 4, cf. a2 and c1). Nevertheless the highest RNA content of all was found in normal tissue: the hymenium. Even in the young pinhead the developing gill tissue contained more RNA than other N1 cells (Fig. 3). Then at maturity RNA content was higher in the hymenial tissue than in the T1 and T2 cells of the same fruit body.

(iv) *Growth measurement*

A comparison of the growth rate of eight tissue cultures with the same number of tissue from the same pileus as the tumour tissue, showed no difference.

### DISCUSSION AND CONCLUSIONS

In order to determine how the tumours arose it is illuminating to examine the normal pattern of differentiation in the fruit body, for comparison with the pattern in tumours.

The general outline of morphogenesis was described in terms of changes in cell shape and size during the formation of the primitive tissues, by Atkinson (1906), Hein (1930), Corner (1935) and Gaumann (1952). All the zones of cell types that would develop into the mature tissues, could be distinguished by the end of the pinhead stage (Fig. 3c). Since the pileus develops endogenously the type of fruit body development was described as hemangiocarpous, unlike that of *Collybia velutipes* where the pileus development is exogenous and the fruit body described as gymnocarpous (Corner, 1935; Gaumann, 1952). Gill tissue developing endogenously does not become exposed until maturity, whereas gill of an endogenous pileus is exposed during development (Fig. 5).

Another aspect of morphogenesis was studied by Bonner, Kane and Levey (1956). They examined the mechanics of growth of the cultivated mushroom by measuring the surface of the fruit body during development and some corresponding changes in histology seen in sections. Growth zones were identified by following the increase in width between equidistant carmine spots painted on the surface of the fruit body at a very early stage. In the stipe most growth occurred in the region next to the cap. In the pileus growth was also uneven, being fastest at the edge and gradually slower at the centre. After the 15–20 mm. stage (in height) in the stalk there was little or no increase in the number of filaments and cells ceased dividing. From the 15–20 mm. stage onwards comparison of dry and wet weights showed that further growth of the fruit body could be attributed to elongation of cells since there was a logarithmic increase in dry matter.

On another level of observation, fundamental cytological studies by Maire (1902), Sass (1928), Colson (1935), and Sarazin (1938) were primarily concerned with the development of the four nuclei resulting from meiosis in the basidium. Evans (1955) paid more attention to the behaviour of nuclei in somatic tissue. Cells undergoing active mitotic activity were most prevalent at the pinhead

---

### EXPLANATION OF PLATES

FIG. 1.—Tumours of the pileus induced by Diesel-oil soot treatment of young fruit bodies at pinhead stage.

FIG. 2.—A test for toxicity; mycelial growth uninhibited by Diesel-oil soot placed at three points around the margin of normal mycelium growing in pure culture on malt agar.

FIG. 3.—Sections through normal fruit bodies at several stages during pinhead differentiation, stained to show the distribution of ribonucleic acid (RNA).

FIG. 4.—Sections through tumour tissue and normal tissue in the mature pileus (Fig. 1) to show the relative distribution of ribonucleic acid (RNA). a: L.S. tumour tip, a1 and a2 showing different staining intensity; b: T.S. tumour, b1 is part of T.S. enlarged; c: L.S. tumour base, c1 is part of L.S. normal pileus enlarged; h: L.S. gill tissue of mature pileus (the photograph shows part of L.S. one gill lamella).

1

2

Hughes.

3

Hughes.

4

stage, but were not found after the early button stage. The present studies confirmed Evans' observations. In addition, from squash preparations it was possible to deduce the regions where divisions occurred in the late pinhead and early button stages. Nuclear divisions were found in non-vacuolated cells of the undifferentiated pinhead, and the cap and upper half of the stipe in the differentiating late pinhead stage (Fig. 3c). In the early button stage (1–2 cm. diameter) nuclear divisions were only found in non-vacuolated cells seen in squash preparations of small pieces of tissue removed from the edge of pileus, the developing

Fig. 5.—Diagram (after Corner, 1935) of longitudinal sections through young fruit bodies showing early stages in two types of Agaric fruit body morphogenesis. 1a, 1b : Hemangiocarpous. 2a, 2b : Gymnocarpous. H : Developing gill tissue.

gill tissue started becoming binucleate, by forming cross walls in the multinucleate cells, by the 1·5–2 cm. diameter stage.

It is interesting to note that the cytological studies are in keeping with the growth measurements and histological studies of Bonner *et al.* (1956). Thus nuclear divisions were found in the regions where increase in the number of filaments and number of cells continued longest. After the 1·5–2 cm. stage mitotic divisions were not found in somatic tissue, a finding which supports the idea that all further growth could be attributed to increase in cell size.

Yet another aspect of morphogenesis contributes to our understanding of the normal pattern of differentiation. During the present studies the growth of the tissues was followed histochemically for the distribution of RNA by studying stained sections. (Although a squash technique would provide the best way of observing the cytology of entire cells, sections were necessary for observing the orientation of tumour cell types to one another and to normal tissue.) Observation of the distribution of RNA could be expected to indicate the metabolically active cells because cytochemical studies on RNA content show a correlation with the level of protein synthesis in tissues (Caspersson, 1947, 1950 ; Brachet, 1950 ; Mirsky, 1951). Also Wade (1955) found that rapid cell division is correlated with a high concentration of RNA rich particles in the cells. Furthermore, *in vitro* biochemical experiments by Gale and Folkes (1954) first provided a direct demonstration that nucleic acids are involved in protein synthesis. (The protein synthesis of fragments of disrupted cells was stopped by the addition of ribonuclease for specific breakdown of RNA. Addition of RNA extracted from the same organism largely restored protein synthesis.) This work has been confirmed by several workers, including Webster and Johnson (1955) and Bridoux and Hanotier (1956). (The latter showed that ribonuclease treatment inhibited incorporation of amino acids into proteins of protoplasts, obtained by dissolving away bacterial cell walls with lysozyme.) These *in vitro* studies are also in agreement with the *in vivo* experiments of Brachet *et al.* (cited by Brachet, 1955, 1958) on living onion root tip cells, where ribonuclease treatment inhibited incorporation of radio-actively labelled amino acids.

In the cultivated mushroom Evans (1955), on the basis of staining reaction with the basic dyes aceto-carmine and pyronin,* concluded that the increased basophilly of the cytoplasm in the Diesel-oil tumour cells compared with normal pinhead cells was due to an increase in RNA synthesis. However, in the present study specific cytochemical staining reactions for RNA showed no increase in the RNA content of the soot-induced T1 cells compared with the N1 cells of the normal pinhead (Fig. 4). (This difference in RNA content between the soot tumour cells and the Diesel-oil tumour cells may be due to a difference in the nature of the tumours, or a difference in technique.) As expected, the soot T2 cells were richer in RNA than N2 cells of the same fruit body, since they were younger cells.

The pattern of the development of cell types to normal maturity and in tumour formation is represented diagramatically in Fig. 6. The N1 and T1 and H cells occupy the deeply staining zones in Fig. 3 and 4, while the N2 and T2 cells are found in the least stained areas.

Since no cytological or histochemical or growth rate differences were found between normal young undifferentiated tissue and the soot induced tumour tissue, it is concluded that the nature of the tumour growth was apparently not abnormal. Their abnormality seems to lie in the continued growth of cells which would normally have ceased doing so, as part of the normal pattern of organisation of the fruit body for its function of spore production.

The studies on tumour production in the cultivated mushroom and the present studies give rise to two views on whch cells gave rise to the tumours :

(a) Evans' (1955) view is that *non-vacuolated cells which had ceased dividing were stimulated to divide again.*—This view is based on the fact that tumours did not appear until a stage in differentiation when nuclear division in cells of the pileus had normally ceased (i.e. after early button stage). Yet it was necessary to start treatment of fruit bodies with Diesel-oil at the pinhead stage since it was

* Although not stated, this was actually pyronin B.

not possible to produce tumours unless treatment was started at pinhead stage. Evans found that despite repeated attempts (with direct application and vapour treatment) to induce tumours by commencing treatment after the pinhead stage no tumours were produced. Also in the present experiment repeated attempts to induce tumours by soot treatment after pinhead stage were unsuccessful. It is more likely that the actively dividing cells of the pinhead were affected by the treatment in such a way as to produce imperfect daughter nuclei incapable of

FIG. 6.—Diagrammatic representation of the pattern of differentiation of cell types to normal maturity and neoplasm formation.

Normal cells :— N1 : not vacuolated ; N2 : vacuolated.
H1 : subhymenium ; H2 : hymenium.
Neoplasm cells :—T1 : not vacuolated ; T2 : vacuolated.

co-operating with other cells in the process of fruit body differentiation. This idea gives rise to the second view, which is put forward here :

(b) *The cells which gave rise to the tumours were ones treated at a metabolically active stage with nuclei undergoing mitosis.*—In the case of the Diesel-oil tumours it was necessary to commence vapour treatment at the pinhead stage and give at least seven days' treatment, so that treatment was actually spread out over several stages of development. However, in the case of the soot-induced tumours there can be no doubt as to which stage in development was treated since only the pinheads were treated during their formation and growth through the casing soil. The active carcinogen known to be present in the soot, benzopyrene, is insoluble in water and non volatile—so treatment of the fruit must have been by direct contact in the casing soil. The evidence for the second view is as follows :

(i) Treatment had to be administered at the pinhead stage when mitotic activity was still going on, or no tumours were produced.

(ii) *Accessibility of susceptible cells.*—The fact that tumours were only formed on the upper surface of the pileus and none on the stipe is interpreted as due to the difference in accessibility of the metabolically active tissues to the carcinogen : —

The absence of tumours of the gill region and the stipe may be attributed to their relative inaccessibility to the carcinogen because of the endogenous development of the pileus. The growth region of the stipe at the stage when mitosis occurs is covered on its outside by tissue which will give rise to the veil (Fig. 3). In animals the fact that most cancers are carcinomas (from surface tissues like epithelium) and that sarcomas (from deep-seated tissues) are least frequent is interpreted as due to the difference in accessibility of the tissues to carcinogenic agents (Cowdry, 1955).

(iii) *It is well known that intermitotic cells are most susceptible to carcinogens* (Cowdry, 1955).—In the mushroom there are two types of cells during differentiation : intermitotics and post-mitotics.

Intermitotics are those cells whose life span depends on the times between division. Their function is to produce new tissue, and reproductive cells. Post-mitotic cells are ones that have finished dividing, and function as units in differentiated tissue until their death.

The intermitotics are of two kinds :

(a) *vegetative intermitotics* (undifferentiated cells) which reproduce their own type and give rise to

(b) *differentiating intermitotics,* so called because, although their nuclei still go through divisions, the cells have developed different specialized properties that distinguish them from undifferentiated cells. The hymenial tissue is an example of this type (also the pileus rim and upper stipe at late pinhead early-button stage). The post-mitotics are cells that no longer undergo mitotic activity. They are the differentiated cells of mature tissues such as stipe and pileus. Normally they are fixed in the post mitotic state, and like the fixed post-mitotics of animals (such as nerve cells) do not revert to mitotic activity, in the differentiated body. Although the mushroom fixed post-mitotic cells and animal post-mitotic cells are capable of mitotic activity in tissue culture, this fact does not affect the present argument because in their natural environment in the differentiated body they are fixed in the undividing condition. (Differentiation in the mushroom is cytoplasmic, since tissue cultures can reproduce the whole organism without variation (Kligman, 1943).)

It appears that in the mushroom the only cells potentially capable of tumour production are the vegetative intermitotics and the differentiating intermitotics, which are the undifferentiated mitotically active cells of the pinhead, the differentiating intermitotics of the pileus rim and top part of the stipe (at the late pinhead and early button stages) and the hymenial tissue. All these tissues except the stipe produced abnormal differentiation in response to carcinogen treatment by Evans (1955). The absence of response in the stipe may have been due to the inaccessibility of the actively mitotic region at its most susceptible stage (late pinhead–early button stage) due to the folding of the developing pileus around the top of the developing stipe. In the Diesel-oil experiment it seems that tumours only arose on fruit bodies where treatment was begun when the mitotically active cells were near the surface of the fruit body. Diesel-oil vapour would not be expected to penetrate deeply because oil and water of the mushroom cells do not mix ! and there is no circulatory system like the blood stream of animals. The position of the soot tumours (Fig. 1) suggests that they were probably due to a small number of intermitotic cells being affected by treatment at the pinhead stage, probably at the rim of the developing pileus (Fig. 3c). The absence of tumours of the hymenium after Diesel-oil treatment can be attributed to the endogenous mode of pileus development so that gill tissue is not exposed until the veil is burst, at a late stage in pileus development. (Although some vapour may penetrate the veil after the early button stage and be responsible for reticulate gill formation.)

(iv) *Latency.*—One of the characteristics of carcinogenic activity in animals is that there is usually a latent period between treatment and the appearance of tumours (Cowdry, 1955).

Latency between the time of treatment and the appearance of the tumours is one of the features noted for the " soot tumours ". Treatment occurred at the pinhead stage, and tumours did not become apparent until early maturity (just after the veil had ruptured), i.e. the tumours were latent during the button stage. (This does not necessarily mean that the cells which gave rise to the tumours were not active during this time. It is probable that the cells responsible were multiplying, possibly from a very small number of cells affected by the carcinogen.)

In the case of the tumours induced by Diesel-oil vapour by Evans (1955), it was stated that at least seven days' treatment was necessary for tumour production, and since treatment was continuous until the tumours appeared it is not possible to distinguish a period of latency between the time of treatment and the appearance of the tumours. Yet such latency may have occurred, since it was not possible to discriminate which cells at what stage of treatment eventually gave rise to the tumours.

In conclusion it is suggested that the most plausible explanation is that the intermitotic cells were affected by carcinogenic treatment, in such a way that imperfect cells were produced lacking in factors, nuclear or cytoplasmic, responsible for sensitivity of cells to cytoplasmic gradients (Mather, 1948) prevailing during normal differentiation, so that they failed to co-operate with other cells in their united efforts towards reproduction of the organism.

Furthermore the delay in appearance of tumours until the button or mature stage, although treatment was necessary at the pinhead stage, is attributed to latency of the affected cells until their activity became morphologically apparent at a later stage.

These ideas would be compatible with the theory of differentiation of cell types put forward by Rusch (1954) that " The final properties of differentiated cells depend on the particular system of reactions nurtured in the first few cellular generations ", i.e. before they become differentiated cell types. Introduction of new factors such as carcinogens into the environment may influence cell development in such a way that some cells lose their capacity to differentiate from the " vegetatively intermitotic " condition and by their active mitoses become tumour-producing cells.

### SUMMARY

1. Diesel-oil soot treatment induced tumours of the pileus.

2. A comparison of the N2 and T2 cells of the same pileus revealed the same number of nuclei.

3. A comparison of the RNA distribution during normal morphogenesis and in the tumour tissue, showed a similarity between the distribution in the tumour tissue and the undifferentiated pinhead.

4. No difference was found between the growth of normal pileus tissue and tumour tissue from the same fruit body in tissue culture.

5. The question about which cells gave rise to the tumours is discussed. The evidence suggests that they were metabolically active intermitotic cells.

The above work was conducted at the Department of Agricultural Botany, University College of Wales, Aberystwyth, under the direction of Professor P. T. Thomas, whose help is gratefully acknowledged. Thanks are also gratefully extended to Professor Alexander Haddow for taking an interest in the use of the cultivated mushroom as an experimental organism, and to the British Empire Cancer Campaign for providing financial support for this work. I am grateful to Mr. K. G. Moreman and staff, particularly Mr. M. J. Docherty, for making photographic enlargements in the Photography Department of the Chester Beatty Research Institute.

### REFERENCES

Atkins, F. C.—(1951) ' Mushroom Growing Today '. London (Faber and Faber Ltd).
Atkinson, G. F.—(1906) *Bot. Gaz.*, **42**, 241, 264.
Beck, S.—(1955) *Rep. Brit. Emp. Cancer Campgn*, **33**, 279.
Bonner, J. T., Kane, K. K. and Levey, R. H.—(1956) *Mycologia*, **48**, 13.
Brachet, J.—(1950) ' Chemical Embryology '. New York (Interscience).—(1958) ' Biochemical Cytology '. New York (Academic Press Inc.).
Bridoux, M. and Hanotier, J.—(1956) *Biochim. biophys. Acta*, **22**, 103.
Caspersson, T.—(1947) *Symp. Soc. exp. Biol.*, No. 1, Nucleic Acids. Cambridge, p. 127. —(1950) ' Cell Growth and Cell Function '. New York (Norton).
Colson, B. (1935) *Ann. Bot., Lond.*, **49**, 1.
Corner, E. J. H. (1935) *Trans. Brit. mycol. Soc.* **19**, 39.
Cowdry, E. V.—(1955) ' Cancer Cells '. London (W. B. Saunders Co.).
Darlington, C. D. and La Cour, L. F.—(1947) ' The Handling of Chromosomes '. London (Allen and Unwin Ltd.).
Evans, H. J.—(1955) ' The Effect of External Agents on Differentiation in the Cultivated Mushroom '. Ph.D. Thesis, University College of Wales.
Gale, E. F. and Folkes, J. P.—(1954) *Nature, Lond.*, **172**, 1223.
Gaumann, E. A.—(1952) ' The Fungi '. London (Hafner Publishing Company).
Glick, D.—(1949) ' Techniques of Histo- and Cytochemistry '. London (Interscience Publishers Ltd.).
Hein, I.—(1930) *Amer. J. Bot.*, **17**, 882.
Henry, S. A.—(1947) *Brit. med. Bull.*, **4**, 389.
Kennaway, E. L. and Heiger, I.—(1930) *Brit. med. J.*, i, 1044.
Kligman, A. M.—(1943) *Amer. J. Bot.*, **30**, 745.
Klioushnikova, E. S.—(1938) *Bull. Soc. Nat. Moscou*, **47**, 30.—(1939) *Ibid.*, **48**, 53.
Komuro, H.—(1931) *Kagaku*, **1**, 182.
Kotin, P., Falk, H. L. and Thomas, M.—(1955) *A.M.A. Arch. Industr. Hlth*, **11**, 113, cited by Lyons (1955).
Lambert, E. B.—(1929) *Science*, **70**, 126.—(1930) *Phytopathology*, **20**, 917.
Levine, M.—(1922) *Amer. J. Bot.*, **9**, 509.
Lindsay, A. J. and Cooper, R. L.—(1951) *Chem. and Ind. (Rev.)*, **1951**, 1066.
Lyons, M. J.—(1955) *Rep. Brit. Emp. Cancer Campgn*, **33**, 279.

MAIRE, R.—(1902) *Bull. Soc. mycol. Fr.*, **19**, Supp., 1.
MATHER, K.—(1948) *Symp., Soc. exp. Biol.*, No. 2, Growth.
MIRSKY, A. E.—(1951) 'Genetics in the 20th Century'. New York (MacMillan), pp. 127–153.
RUSCH, H. P.—(1954) *Cancer Res.*, **14**, 407.
SARAZIN, A.—(1938) *C.R. Acad. Sci., Paris*, **206**, 275.
SASS, J. E.—(1928) *Pap. Mich. Acad. Sci.*, **9**, 289.
SINDEN, J. W.—(1937) cited by Kligman (1943).
SWIFT, H.—(1955) In 'The Nucleic Acids', Vol. II, ed. Chargaff and Davidson. New York (Academic Press), pp. 51–92.
THOMAS, P. T.—(1951) *Rep. Brit. Emp. Cancer Campgn*, **29**, 286.
*Idem* AND EVANS, H. J.—(1954) *Ibid.*, **32**, 428.
TOMKINS, R. G.—(1932) *Trans. Brit. mycol. Soc.*, **17**, 150.
TURCHINI, J., CASTEL, P. AND VAN KIEN, L. K.—(1948) *C.R. Soc. Biol., Paris*, **142**, 1277.
WADE, H. E.—(1955) *Nature, Lond.*, **176**, 310.
WEBSTER, G. C. AND JOHNSTON, M. P.—(1955) *J. biol. Chem.*, **217**, 641.
YAMAGIWA, K. AND ICHIKAWA, K.—(1916) *Mitt. med. Fak. Tokio*, **15**, 295.